# Value of preoperative computed tomography for meso-Rex bypass in children with extrahepatic portal vein obstruction

**DOI:** 10.1186/s13244-021-01057-8

**Published:** 2021-07-27

**Authors:** Huiying Wu, Ning Zhou, Lianwei Lu, Xiwen Chen, Tao Liu, Binbin Zhang, Hongsheng Liu, Zhe Wen

**Affiliations:** 1 Department of Radiology, Guangzhou Women and Children’s Medical Center, 9 Jinsui Road, Guangzhou, 510623 Guangdong China; 2 Department of Pediatric Surgery, Guangzhou Women and Children’s Medical Center, 9 Jinsui Road, Guangzhou, 510623 Guangdong China

**Keywords:** Rex shunt, Cavernous transformation, Pediatrics, Computed tomography, Portal hypertension

## Abstract

**Background:**

Extrahepatic portal vein obstruction (EHPVO) is the most important cause of hematemesis in children. Intrahepatic left portal vein and superior mesenteric vein anastomosis, also known as meso-Rex bypass (MRB), is becoming the gold standard treatment for EHPVO. We analyzed the value of preoperative computed tomography (CT) in determining whether MRB is feasible in children with EHPVO.

**Results:**

We retrieved data on 76 children with EHPVO (50 male, 26 female; median age, 5.9 years) who underwent MRB (*n* = 68) or the Warren procedure (*n* = 8) from 2013 to 2019 and retrospectively analyzed their clinical and CT characteristics. The Rex recess was categorized into four subtypes (types 1–4) depending on its diameter in CT images. Of all 76 children, 7.9% had a history of umbilical catheterization and 1.3% had leukemia. Sixteen patients (20 lesions) had associated malformations. A total of 72.4% of Rex recesses could be measured by CT, and their mean diameter was 3.5 ± 1.8 mm (range 0.6–10.5 mm). A type 1, 2, 3, and 4 Rex recess was present in 9.2%, 53.9%, 11.8%, and 25.0% of patients, respectively. MRB could be performed in patients with types 1, 2, and 3, but those with type 4 required further evaluation. The sensitivity, specificity, positive predictive value, negative predictive value, and diagnostic accuracy of CT were 100%, 83.8%, 42.1%, 100%, and 85.5%, respectively.

**Conclusions:**

Among the four types of Rex recesses on CT angiography, types 1–3 allow for the performance of MRB.

## Key points

- The Rex recess can be categorized into four subtypes depending on its diameter.
- The sensitivity, specificity, PPV, NPV, and diagnostic accuracy of preoperative CT for MRB were 100%, 83.8%, 42.1%, 100%, and 85.5%, respectively.
- A type 4 Rex recess requires further examination before MRB.

## Background

Extrahepatic portal vein obstruction (EHPVO) is defined as thrombosis of the extrahepatic portal vein (PV) with or without extension to the intrahepatic PVs [1]. It is the cause of portal hypertension in 70% of pediatric patients and the most common cause of upper gastrointestinal bleeding in children [2, 3]. Underlying etiologies of PV thrombosis include sepsis, dehydration, intra-abdominal/pelvic infection, omphalitis, umbilical vein catheterization, a hypercoagulable state, biliary atresia, and chronic liver disease [4]. In up to 50% of children and young adults with EHPVO, the underlying etiology of the PV thrombosis remains unknown [2, 5]. Patients may present with splenomegaly, ascites, encephalopathy, or cardiopulmonary complications.

**Fig. 1:**
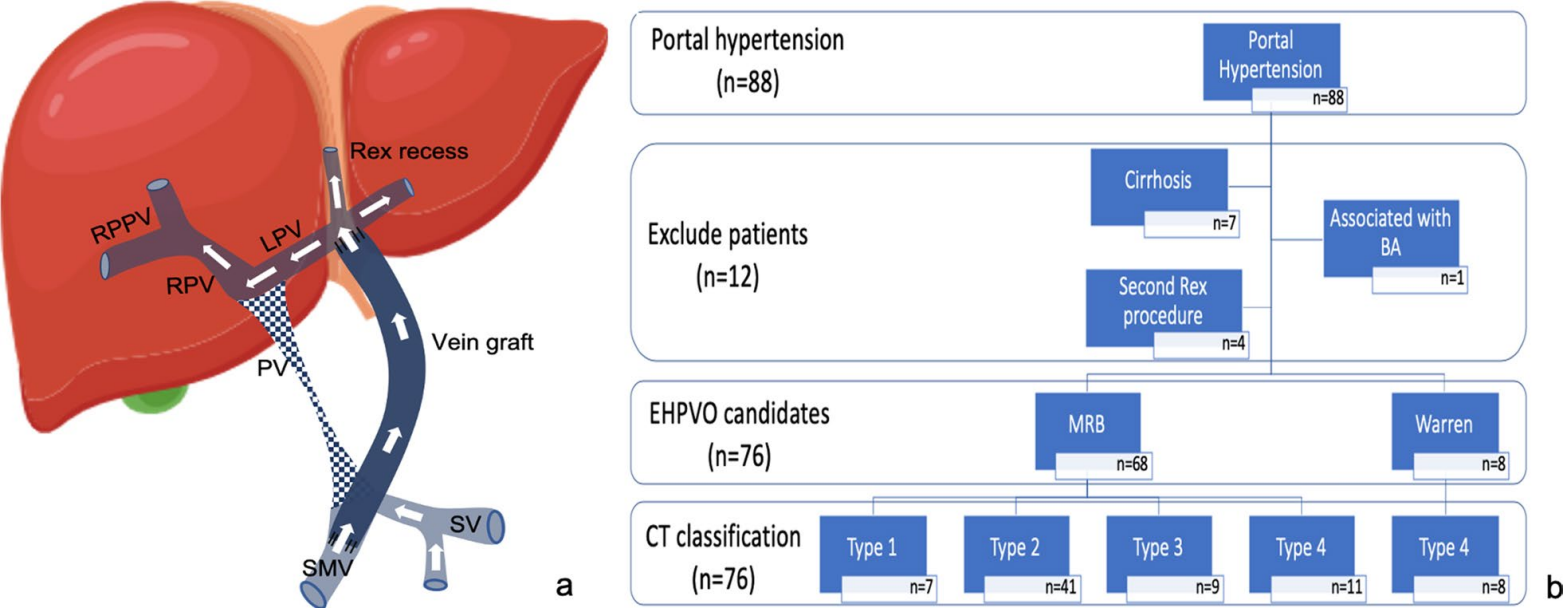
**a** Diagram of the meso-Rex bypass procedure. **b** Children with portal hypertension (October 2013 to December 2019). Abbreviations: SMV, superior mesenteric vein; SV, splenic vein; PV, portal vein; LPV, left portal vein; RPV, right portal vein; RPPV, right posterior portal vein; BA, biliary atresia; EHPVO, extrahepatic portal vein obstruction; CT, computed tomography; and MRB, meso-Rex bypass

The Rex recess is the remnant of the embryonic umbilical vein. It is the space between hepatic segments III and IV, where the intrahepatic left PV (LPV) is conveniently placed for mesentericoportal anastomosis to restore hepatopetal flow. Meso-Rex bypass (MRB), also known as the Rex shunt (Fig. 1a), is the definitive treatment for EHPVO [6]. This procedure restores physiological portal liver reperfusion via a venous autograft connection from the superior mesenteric vein (SMV) to the intrahepatic LPV. MRB is therefore the gold standard treatment in children with favorable anatomy.

Preoperative imaging is essential because it provides anatomical information for surgical planning and excludes diseases for which MRB is not recommended; it is especially useful to determine the patency and size of the SMV and Rex recess. Preoperative ultrasound with color Doppler has been utilized to examine the patency of the LPV and SMV for MRB [7, 8]. However, this examination technique is difficult because of the low flow velocity and small vascular caliber in pediatric patients. Magnetic resonance imaging (MRI) and computed tomography (CT) have been described as effective modalities in the evaluation of children with EHPVO [9, 10]. They can provide an anatomical road map of the splanchnic and portal venous anatomy and create three-dimensional reconstructions to display spatial relationships. MRI has higher contrast resolution, but the longer acquisition times make the sequences sensitive to motion. It is also difficult for children to breath-hold during MRI. CT has higher spatial resolution, and CT angiography has been described as an effective modality. Notably, however, Superina et al. [6] successfully performed MRB in patients whose LPV was not visible by preoperative imaging. Bertocchini et al. [11] reported that wedged hepatic vein portography (WHVP) was an effective tool for preoperative assessment of the Rex recess. WHVP involves the performance of venous puncture for catheter access to the liver and can provide information on Rex recess patency. However, WHVP is an invasive examination that requires general anesthesia and endotracheal intubation.

No previous CT study or published classification has addressed the accuracy of using the characteristics of the Rex recess compared with the findings of direct visualization to identify pediatric patients with EHPVO who are good candidates for MRB. Our goal was to elucidate the technique and value of preoperative CT in evaluating the feasibility of MRP in children with EHPVO. Further, we calculated the proportion of patients whose LPV was not visible by preoperative imaging but who successfully underwent MRB, and we proposed follow-up management for these patients.

## Methods

### Clinical data

A database of children aged ≤ 14 years (range 0.7–14 years) with EHPVO was reviewed to identify MRB candidates who had undergone preoperative CT imaging at our institution from 2013 to 2019 (Fig. 1b). The inclusion criteria were noncirrhotic prehepatic portal hypertension caused by extrahepatic PV obstruction, which was defined by classic symptoms of portal hypertension (esophageal varices, splenomegaly, and hypersplenism with or without hyperammonemia, coagulopathy, or ascites); no evidence of associated liver disease (normal liver function test results and normal appearance); an abnormal PV trunk on imaging; and cavernous venous collaterals at the porta hepatis. The exclusion criteria were hepatic and post-hepatic portal hypertension, cirrhosis, and a history of having undergone MRB. In total, 76 patients were finally included in the study.

The clinical data included the age at surgery, sex, disease course, symptoms, associated malformations, history of umbilical vein intubation, and treatment history. All procedures were performed by the senior surgeon (Z.W.) or under his direct supervision. The left internal jugular vein/left gastric (coronary) vein was used as the vein graft. The Rex recess was examined by direct intraoperative observation or direct intraoperative Rex recess angiography. If the Rex recess was aplastic, selective portosystemic shunt–distal splenorenal shunt (Warren procedure) was considered. The Warren procedure is a nonphysiological and nonpermanent [1, 12] shunt surgery that can maintain some portopetal flow and avoid encephalopathy. A successful Rex operation is indicated by disappearance of the clinical symptoms, normalization of the laboratory indicators related to hypersplenism, and a smooth anastomotic opening on ultrasound examination 3 days postoperatively. All 76 children were followed up after surgery. Follow-up involved assessment for any recurrence of clinical symptoms, assessment of laboratory test results, and performance of regular ultrasound and CT examinations. Nine (9/76, 11.8%) patients had anastomotic stenosis. The follow-up time ranged from 7 days to 6.4 years (median, 1.7 years).

### Examination methods

All 76 patients underwent plain and contrast-enhanced abdominal CT. Before the examinations, the children fasted for 3–4 h, and those aged ≤ 5 years were orally administered chloral hydrate (0.5 ml/kg) for sedation. A 64-slice spiral CT scanning protocol was used with the following settings: tube voltage, 120 kV; automatic current conditions; thickness and layer spacing, 0.8 mm; matrix, 512 × 512; and standard algorithm reconstruction image with 1-mm layer thickness. CT angiography was performed with 2 ml/kg of contrast medium (Ultravist 300) during the arterial phase, portal venous phase, and delayed phase at 23 s, 45–52 s, and 90 s, respectively. Image post-processing included multiplanar reformation, maximum intensity projection, shaded surface display, and volume rendering. The duration from CT to MRB ranged from 1 to 194 days (median, 33 days).

### Image analysis

The CT images were reassessed by two radiologists with 6 and 10 years of experience in pediatric radiology, respectively, who were blinded to the surgery. The radiologists reached a consensus regarding the imaging results.

Gastric fundic varices, the left gastric (coronary) vein [13], and cavernous transformation of the PV were defined as common portocaval shunts. Other portocaval shunts were defined as rare shunts. We also evaluated other abdominal findings including gallstones, the biliary tree, and ascites.

**Fig. 2:**
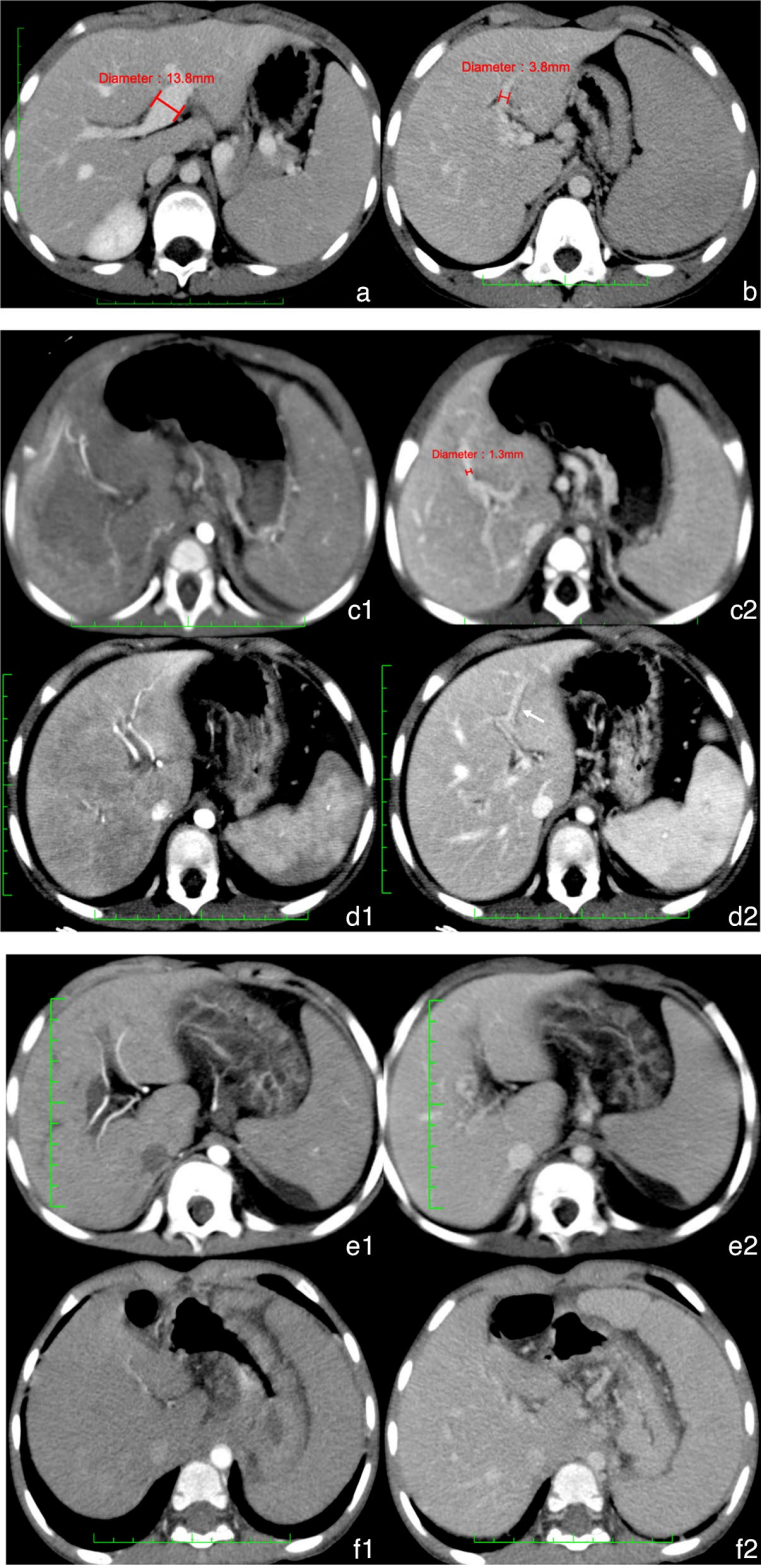

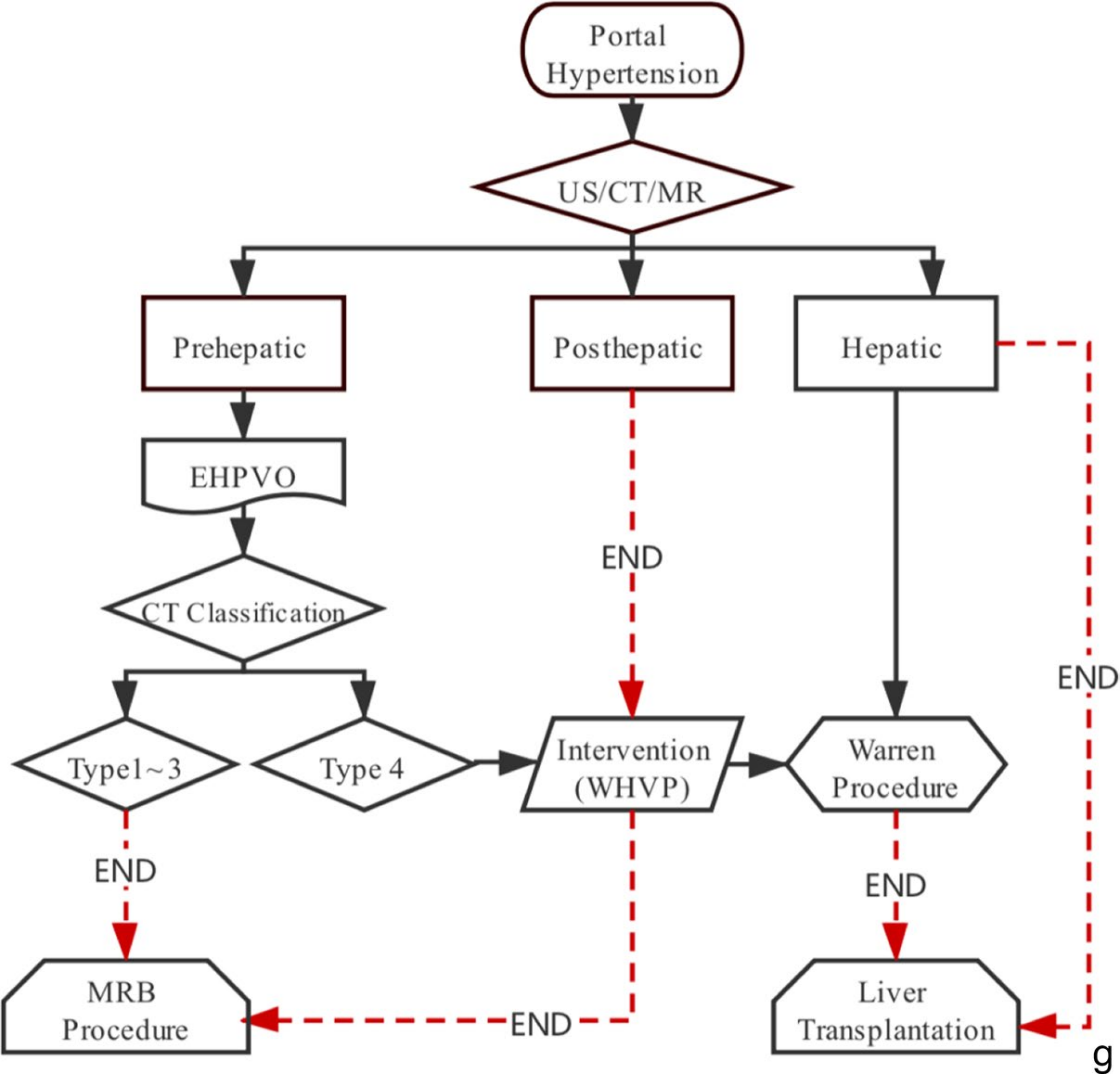
Computed tomography classification of the Rex recess. **a** Type 1. The Rex recess is widened, and the diameter is ≥ 5 mm (13.8 mm in this figure). **b** Type 2. The Rex recess is clear and can be measured, and the diameter is ≥ 2 to < 5 mm (3.8 mm in this figure). **c**, **d** Type 3. The Rex recess is faintly visible and < 2 mm in diameter, or the border with the side branches is unclear, but the segment III branch can be distinguished by computed tomography. **c1**, **c2** The diameter of the Rex recess is < 2 mm (1.3 mm in delayed phase imaging). **c2** Note the thickened left hepatic artery in the arterial phase imaging of **c1**. **d1, d2** The Rex recess cannot be distinguished from the thickened left hepatic artery (**d1**), but the branch of segment III can be found in **d2**. **e**, **f** Type 4. The Rex recess cannot be distinguished by computed tomography. **e1**, **e2** The Rex recess cannot be distinguished from the thickened left hepatic artery and cannot be confirmed in the branch of segment III. **f1**, **f2** Complete loss of vascular landmarks of the Rex recess. **g** Diagram of the meso-Rex bypass procedure. Abbreviations: US, ultrasound; CT, computed tomography; MR, magnetic resonance imaging; EHPVO, extrahepatic portal vein obstruction; MRB, meso-Rex bypass; and WHVP, wedged hepatic vein portography

The maximum diameters of the SMV, splenic vein (SV), spleen, and Rex recess were measured twice in the portal phase, and the mean was used for analysis. The spleen diameter was measured in the coronal plane, and all vascular diameters were measured in the axial plane. We retrospectively assessed the CT findings of children with EHPVO and herein propose a system for further classification of the Rex recess according to the CT features. The CT pattern of the Rex recess was categorized into four subtypes:

- Type 1 (Fig. 2a): The diameter of the Rex recess is ≥  5 mm.
- Type 2 (Fig. 2b): The diameter of the Rex recess is 2 to < 5 mm.
- Type 3 (Fig. 2c, d): The Rex recess is faintly visible and < 2 mm in diameter, or the border with the side branches is unclear but segment III can be distinguished.
- Type 4 (Fig. 2e, f): The Rex recess cannot be distinguished.

We calculated the accuracy, sensitivity, specificity, positive predictive value (PPV), and negative predictive value (NPV) of CT to identify the feasibility of MRB compared with laparotomy.

A diagram of the pediatric portal hypertension procedure is shown in Fig. 2g.

### Statistical analysis

The statistical analysis was performed using SPSS 26.0 (IBM Corp.). Statistical significance was achieved at a 0.05 level.

The categorical variables (sex, associated malformations, intraductal bile duct dilatation, common portocaval shunt, rare portocaval shunt, and ascites) were analyzed by Pearson’s Chi-square test. The Kolmogorov–Smirnov normality test was used to assess continuous variables (age at surgery, disease course, and diameters of Rex recess, SMV, SV, and spleen). The continuous variables were not normally distributed, and the Kruskal–Wallis test was used.

Successful MRB was considered a true positive, and aplasia of the Rex recess was considered a true negative. The sensitivity, specificity, PPV, NPV, and accuracy were calculated.

Next, we used binary logistic regression to analyze multiple variables (sex, age at surgery, disease course, and diameters of Rex recess, SMV, SV, and spleen) for their effect on predicting MRB surgery.

## Results

### Patient characteristics

MRB was attempted in 76 children with EHPVO. Six (6/76, 7.9%) patients had a history of umbilical catheterization, 1 (1/76, 1.3%) had a previous diagnosis of leukemia, and 69 (69/76, 90.8%) had an unknown cause of EHPVO. The initial symptom was variceal hemorrhage in 58 (58/76, 76.3%) patients, bloody/black stools in 31 (31/76, 40.8%), and splenomegaly/hypersplenism in 23 (23/76, 30.2%). Sixty-two (62/76, 81.6%) children underwent gastroscopy, including 30 (30/62, 48.4%) with mild, 10 (10/62, 16.1%) with moderate, and 22 (22/62, 35.5%) with severe esophageal varices. The mean platelet count was 106 ± 60 × 10^9^ (range 22–290 × 10^9^; reference range 140–440 × 10^9^). One patient underwent surgical repair of splenic rupture, two underwent the Warren procedure, and three underwent splenectomy before CT.

Sixteen patients (20 lesions) had associated malformations. Seven patients had common bile duct cysts (six postoperatively, one preoperatively), four had congenital heart disease, three had renal malformations (two horseshoe kidney, one polycystic kidney), two had intestinal malformations (hypertrophic pyloric obstruction, intestinal malrotation), two had spinal deformity, one had gallstones, and one had an inguinal hernia. Sixty-eight patients successfully underwent the Rex procedure, and eight underwent the Warren procedure when the surgeon found that the Rex recess was dysplastic.

The continuous variables were not normally distributed (Table 1). Children who underwent the Warren procedure had a higher proportion of malformations than children who underwent MRB (*p* = 0.006). There were no other significant differences between MRB and the Warren procedure.

**Table 1:** Clinical data and imaging features of 76 children with EHPVO

	MRB	Warren	Total	*p*
No	89.5% (68)	10.5% (8)	100 (76)	NA
Age (year)^	5.0 (3.0)	4.0 (5.3)	5.0 (3.0)	0.306
Gender (*F*%)^∇^	35.3% (24)	25.0% (2)	34.2% (26)	0.562
The course of the disease (D)^	300.0 (983.8)	713.5 (1454.5)	300 (1075)	0.786
Interval time^	17 (21)	14 (13)	17 (21)	0.588
Associated malformations (+)^∇^	19.1% (13)	62.5% (5)	(18)	0.006*
Rex recess^	3.0/2.0 (2.0)	NA	3.0 (2.0)	NA
The diameter of SMV (mm)^	6.4 (2.4)	7.0 (1.9)	6.4 (2.4)	0.418
The diameter of SV (mm)#^	5.0 (2.2)	5.9 (3.7)	5.1 (2.1)	0.551
Diameter of spleen (cm)^	13.2/4.2 (4.2)	13.1/4.0 (4.0)	13.1 (4.2)	0.543
Esophageal-gastric varices (+)^∇^	97.1% (66)	100% (8)	97.4% (74)	0.623
Cavernous transformation of PV (+)^∇^	92.6% (63)	75% (6)	90.8% (69)	0.103
Uncommon portocaval shunt (+)^∇^	26.5% (18)	12.5% (1)	25% (19)	0.388
Dilatation of bile duct (+)^∇^	13.2% (9)	12.5% (1)	13.2% (10)	0.954
Ascites (+)^∇^	19.1 (13)	12.5% (1)	18.4% (14)	0.648

^Mann–Whitney U test

^∇^Pearson’s Chi-square test (Fisher’s exact test)

*Differences are statistically significant

Age (age at surgery), disease course, interval time (duration from surgery to computed tomography angiography), and diameter are shown as median (interquartile range)

*EHPVO* extrahepatic portal vein obstruction, *MRB* meso-Rex bypass, *SMV* superior mesenteric vein, *SV* splenic vein, NA

### Imaging findings and visualization of Rex recess

**Fig. 3:**
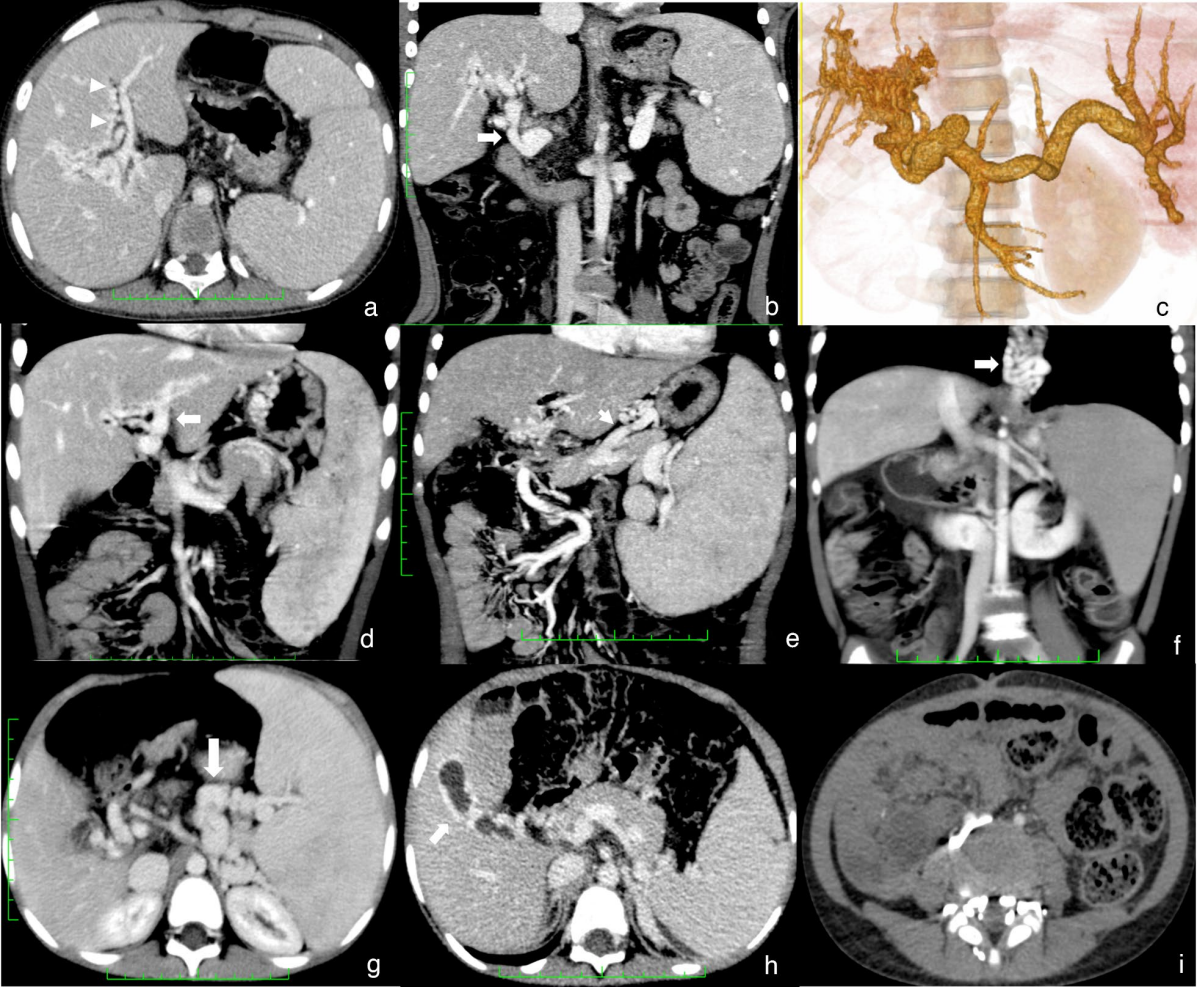
Imaging manifestations of extrahepatic portal vein obstruction in children. **a**–**c** Cavernous transformation (white triangle) in the Rex recess. **d** Cavernous transformation of the portal vein (white arrow). **e** Gastric coronary varices. **f** Esophageal-gastric varices (white arrow). **g** Splenorenal shunt (white arrow). **h** Gallbladder varices. **i** Paravertebral varices

**Fig. 4:**
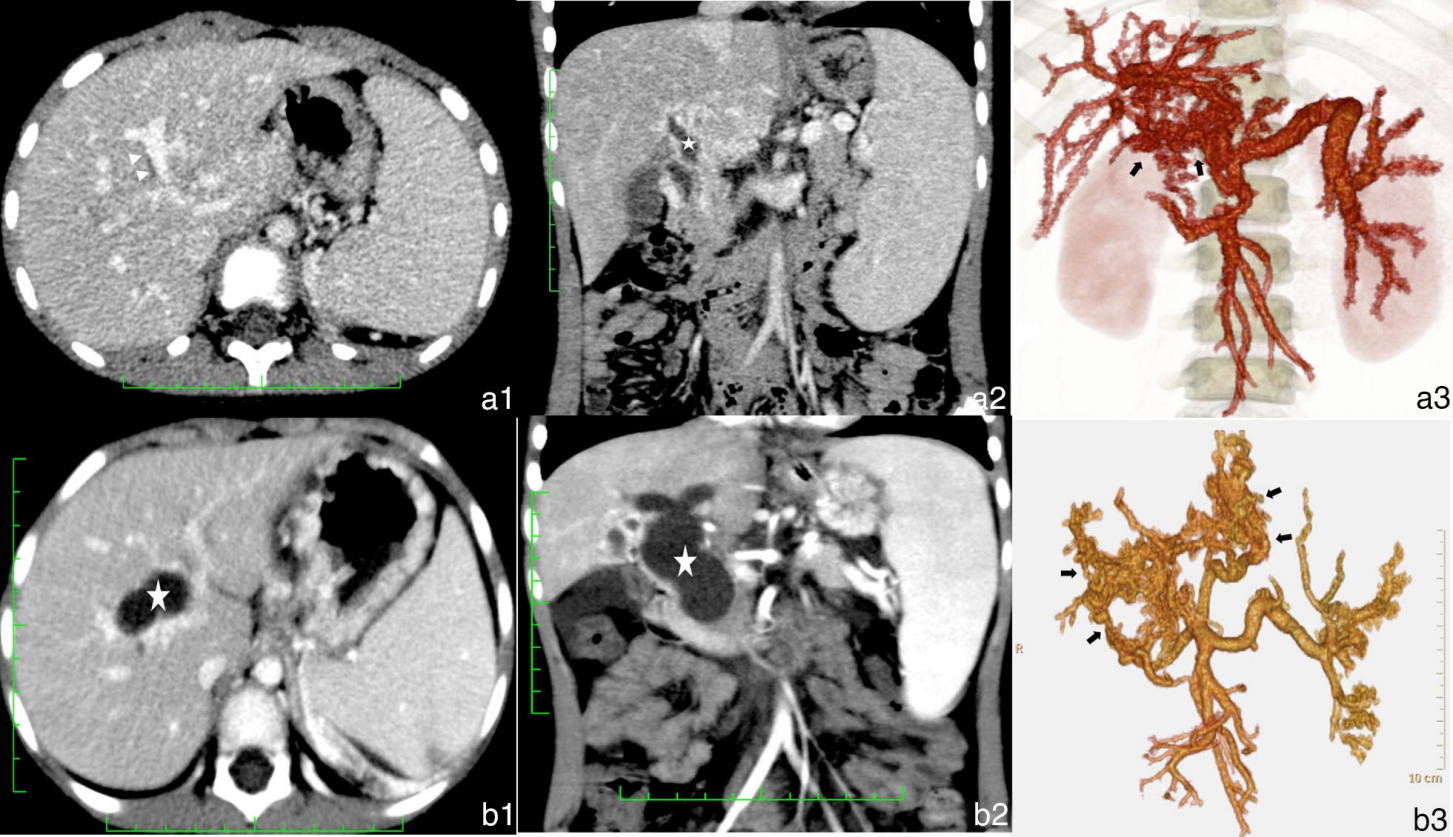
**a** Male patient aged 7 years. Extrahepatic portal vein obstruction (EHPVO) combined with bile duct dilation. **a1, a2** Bile duct dilation (white triangle) next to the portal vein and dilation of the common hepatic duct (white star). **a3** Volume rendering (VR) showed cavernous transformation (black arrow). **b** Female patient aged 5 years. EHPVO combined with a common bile duct cyst. **b1, b2** Dilation of intrahepatic bile duct and common bile duct (white star). **b3** VR showed cavernous transformation (black arrow)

The CT manifestations of EHPVO were the formation of cavernous collaterals, cavernous transformation of the PV (Fig. 3b–d), portal hypertension (Fig. 3a, c), portosystemic shunt, biliary dilatation (Fig. 4a), and ascites (Table 2). The portosystemic shunts included esophageal and gastric varices (Fig. 2d, e) and other rare portocaval shunts (Fig. 3d–f), such as splenorenal shunts (Fig. 2g), gallbladder vein dilation (Fig. 3h), and paravertebral varices (Fig. 3i). Additionally, 1.3% (10/76) of patients had common bile duct cysts (Fig. 4b).

**Table 2:** Binary logistic regression of multivariate analysis for meso-Rex bypass

	Chi-square	*df*	Sig.
Omnibus tests of model coefficient			
Step	17.430	1	.000
Block	17.430	1	.000
Model	17.430	1	.000

	*B*	SE	Wald	*df*	Sig.	Exp (B)	95% C.I. for EXP (B)	
							Lower	Upper
Variables in the equation								
Rex recess	− 22.863	2336.420	.000	1	.992	.000	.000	
Constant	− .773	.494	2.454	1	.117	.462		

	Score	*df*	Sig
Variables not in the equation			
Sex (F)	.101	1	.750
Age (year)	.513	1	.474
Interval time (D)	1.291	1	.256
The course of the disease (D)	.620	1	.431
The diameter of SMV (mm)	1.705	1	.192
The diameter of SV	.589	1	.443
Diameter of spleen (cm)	.281	1	.596
Overall statistics	6.082	7	.530

*df* degrees of freedom, *Sig.* significance, *SE* standard error, *CI* confidence interval, *F* female, *SMV* superior mesenteric vein, *SV* splenic vein

The mean SMV diameter was 6.9 ± 2.2 mm (range 2.5–13.7 mm). The mean diameter of the SV and spleen was 5.6 ± 2.0 mm (range 2–12 mm) and 13.2 ± 3.3 cm (range 2.8–21.9 cm), respectively.

No patients had an intraluminal PV thrombus. We did not measure the RPV because it was difficult to distinguish the RPV from the collateral veins.

In 55 (55/76, 72.4%) patients, the mean diameter of the Rex recess was 3.5 ± 1.8 mm (range 1.0–11.0 mm). Binary logistic regression analysis showed that only the diameter of the Rex recess had statistical significance for prediction of MRB (*p* = 0.000): Logistic (*P*) =  − 0.773 + (− 22.863) × Rex recess (Table 2).

In 48 (48/76, 63.2%) patients, the diameter was > 2 mm. Two patients showed only the branch of segment III, and 19 patients showed no sign of the LPV. According to our CT definition, type 1 was present in 7 patients (Fig. 2a), type 2 in 41 (Fig. 2b), type 3 in 9, and type 4 in 19 (Fig. 2e, f). In seven (7/9, 77.8%) patients with type 3, the median (interquartile range) diameter of the Rex recess was 1.4 (0.4) mm (Fig. 2c). In two (2/9, 22.2%) patients, the Rex recess could not be distinguished from the thickened artery or collateral vessel, but segment III could be found (Fig. 2d).

A significant difference was found in the diameter of the SV (*p* = 0.018). Further two–two comparisons showed that the SV diameter was shorter in type 4 than type 1 (*p* = 0.030). Categorical variables were analyzed by Pearson’s Chi-square test, and there was no significant difference among them (Table 3).

**Table 3:** Computed tomography findings and clinical data of pediatric patients with EHPVO

	Type 1	Type 2	Type 3	Type 4	Total	*p*
No.	9.2% (7)	53.9% (41)	11.8% (9)	25.0% (19)	100% (76)	NA
Age (year)^	5.0/4.0 (3.0–12.0)	5.0/3.5 (1.3–13.0)	6.0/2.5 (0.7–14.0)	4.0/3.0 (1.3–14.0)	5.0/3.0 (0.7–14)	0.221
Gender (*F*%)^∇^	28.6% (2)	34.1% (14)	66.7% (6)	21.1% (4)	34.2% (26)	0.123
Course of disease (Y)^	2.0/4.8 (0.2–5.0)	0.7/1.9 (0.0–8.0)	1.0/4.5 (0.0–6.0)	1.0/3.9 (0.0–8.0)	0.8/2.9 (0.0–8.0)	0.528
Interval time ^	7/120 (2–35)	17/16 (1–176)	27/59 (5–168)	14/31 (7–194)	17/21 (1–194)	0.277
Associated malformations (+)^∇^	28.6% (2)	17.1% (7)	11.1% (1)	42.1% (8)	25% (19)	0.142
Rex recess (mm)^	6.5/3.5 (5.0–10.5)	2.9/1.3 (2.0–4.8)	1.4/0.4 (0.6–3.0)	NA	3.0/2.0 (0.6–10.5)	0.000*
Diameter of SMV (mm)^	7.3/6.5 (5.9–13.7)	7.0/2.7 (3.5–12.4)	6.0/1.5 (4.7–11.2)	6.4/3.1 (2.5–8.5)	6.4/2.4 (2.5–13.7)	0.161
Diameter of SV (mm)^	6.7/3.7 (5.2–11.7)^a^	5.0/2.1 (1.9–11.3)	4.9/1.6 (1.8–7.4)	4.8/2.0 (2.5–9.3)^b^	5.1/2.2 (1.8–11.7)	0.027*
Diameter of spleen (cm)^	15.3/3.3 (12.1–18.0)	13.0/4.4 (2.8–21.9)	11.7/4.3 (8.1–15.4)	13.2/4.2 (8.8–17.8)	13.1/4.1 (2.8–21.9)	0.129
Esophageal-gastric varices (+)^∇^	100% (7)	95.1% (39)	100% (9)	100% (19)	97.4% (74)	0.625
Cavernous transformation of PV (+)^∇^	100% (7)	92.7% (38)	100% (9)	78.9% (15)	90.8% (69)	0.173
Uncommon portocaval shunt (+)^∇^	14.3% (1)	29.3% (12)	33.3% (3)	15.8% (3)	25% (19)	0.568
Dilatation of bile duct (+)^∇^	14.3% (1)	9.8% (4)	22.2% (2)	15.8% (3)	13.2% (10)	0.757
Ascites (+)^∇^	NA (0)	17.1% (7)	44.4% (4)	15.8% (3)	18.4% (14)	0.123

^Kruskal–Wallis test

∇Pearson’s Chi-square test (Fisher’s exact test)

^#^Post hoc test is statistically significant (Bonferroni correction)

^*^Differences are statistically significant

Age (age at surgery), disease course, interval time (duration from surgery to computed tomography angiography), and diameter are shown as median/interquartile range (range)

^a,b^The difference between these two groups is statistically significant, and the incidence is *a* > *b*

*EHPVO* extrahepatic portal vein obstruction, *SMV* superior mesenteric vein, *SV* splenic vein, *PV* portal vein, NA

### Value of CT in evaluation of Rex recess

In all 76 (76/76, 100%) patients, the SMV could be visualized. In 73 (73/76, 96.1%) patients, the SV was visualized; the remaining 3 patients had previously undergone splenectomy.

All patients with a type 1, 2, or 3 Rex recess successfully underwent MRB. Among 19 patients with type 4, 57.9% (11/19) were proven suitable to undergo MRB; the remaining 42.1% (8/19) were found to have Rex recess aplasia and underwent the Warren procedure. We assigned the patients with a type 1, 2, and 3 Rex recess into the MRB group and those with type 4 into the potential MRB group. The sensitivity, specificity, PPV, NPV, and diagnostic accuracy of CT were 100%, 83.8%, 42.1%, 100%, and 85.5%, respectively.

### Impact of time and treatment on CT classification

**Fig. 5:**
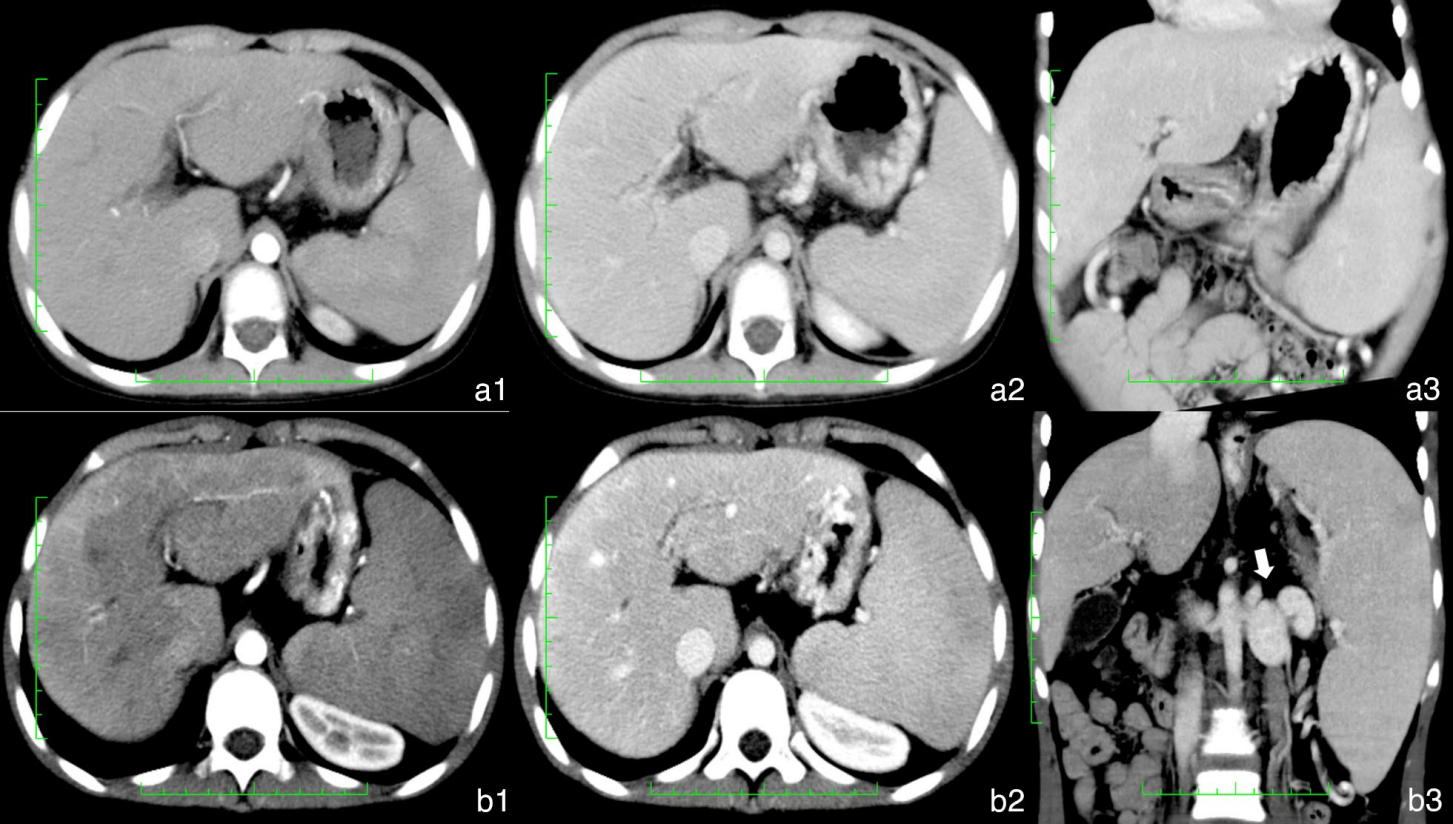
Male patient aged 6 years 4 months with a 3-year history of repeated hematemesis. **a** Complete loss of vascular landmarks of the Rex recess (type 4). The patient underwent the Warren procedure. **b** Computed tomography angiography after 56 months. Complete loss of vascular landmarks of the Rex recess. **b3** The spleen–kidney vein anastomosis and the spleen were larger than before

**Fig. 6:**
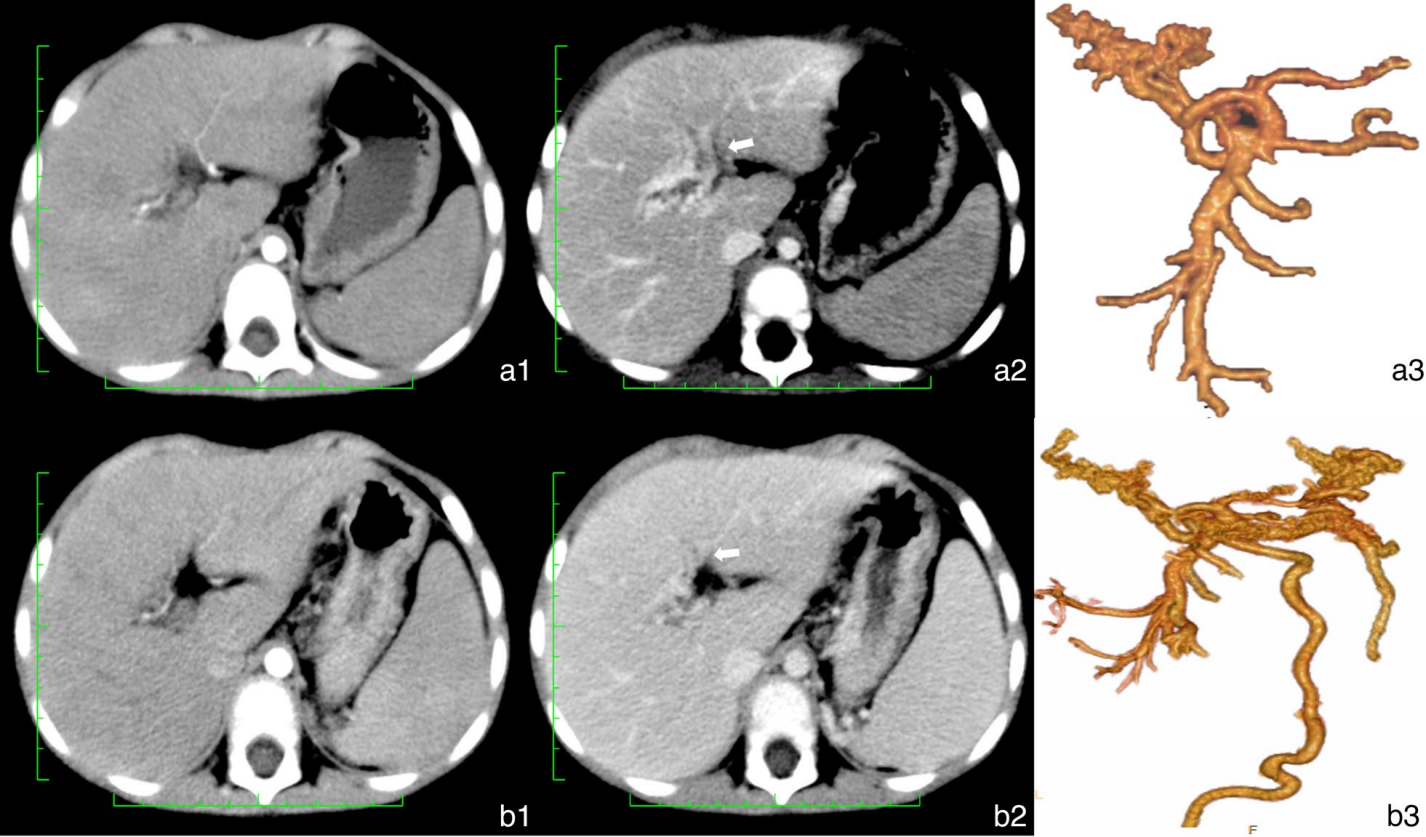
Male patient aged 2 years 6 months with a 6-month history of black stool. **a** The Rex recess was faintly visible and the diameter of the Rex recess (white arrow) was 1.8 mm (type 3). **b** Computed tomography angiography after 18 months. Complete loss of vascular landmarks of the Rex recess (white arrow), which was type 4. **b3** Volume rendering showed an increase in cavernous transformation

Six patients (Table 4) underwent CT re-examination before MRB (mean, 2.8 years). As the disease progressed, the spleen diameter increased. Five patients had no change in their CT classification, and one of them underwent the Warren procedure (Fig. 5). One patient changed from type 3 to 4 with conservative treatment (Fig. 6).

**Table 4:** Re-examination CT angiography before MRB in six patients

No.	Gender	Age*	Interval time (*Y*)*	Associated malformations	CT type	Rex recess (mm)	SMV (mm)	SV (mm)	Spleen (cm)	Treatment
1	M	4Y	1.5	NA	3	1.7	5.5	2.7	12.3	Conservative treatment
					4	NA	5.5	3.7	15.0	
2	M	6Y	4.8	NA	3	NA	5.7	3.2	9.4	Conservative treatment
					3	NA	4.7	3.2	12.7	
3	F	8Y	1.3	NA	2	4.1	5.6	4.1	12.0	Conservative treatment
					2	4.5	5.8	4.6	13.3	
4	M	10Y	4.7	NA	4	NA	6.0	7.4	12.1	Warren procedure
					4	NA	4.3	9.3	17.7	
5	M	4Y	1.4	Fallot 4	4	NA	4.8	3.8	12.4	Conservative treatment
					4	NA	5.4	5.8	13.1	
6	M	4Y	2.8	NA	2	2.3	3.3	2.1	10.0	Conservative treatment
					2	2.1	3.5	2.2	13.2	

*MRB* meso-Rex bypass, *M* male, *F* female, *Age age at surgery, *Interval time examination time between two CT angiography procedures, *CT* computed tomography, *SMV* superior mesenteric vein, *SV* splenic vein, *NA*

## Discussion

In the present study, EHPVO was the most important cause of portal hypertension, and the patients’ age at the time of diagnosis and surgery was slightly older because of the long disease course. Portal hypertension was caused by EHPVO in 90.9% of children, by biliary atresia in 1.1%, and by cirrhosis in 8.0%. According to the literature, EHPVO is typically diagnosed between the ages of 2 and 4 years [7]; the median age in our study was 5.9 years. Because of the unbalanced level of medical development in China, not all regions can perform MRB for children, and the history of umbilical vein intubation was unclear in most children of our study. Notably, of seven children with combined common bile duct cysts, six patients’ cysts developed within a few years after common bile duct surgery. However, the relationship between the history of common bile duct surgery and EHPVO remains unclear. All of these data suggest that the condition of the PV should be carefully evaluated at the time of the first diagnosis of a choledochal cyst and after completion of surgical treatment of the choledochal cyst. Additionally, the children with Rex dysplasia in our study had a higher incidence of combined malformations. However, we were unable to identify the specific types of deformities because of the small sample.

CT is an effective modality in the evaluation EHPVO [14, 15] with the characteristic findings of cavernous transformation and portal hypertension. No thrombi were observed because most patients had a long history. Gastroesophageal varices and the left gastric (coronary) vein occurred in 97.4% of children, followed by cavernous transformation of the PV in 90.8%. This collateral was cavernous transformation of the extrahepatic PV that meandered into the hilar region and supplied the right liver, and it was difficult to distinguish from the trunk of the RPV. The rare portocaval shunts were splenorenal veins, gallbladder veins, and paravertebral veins. In the ideal presurgical candidate for MRB, both the intrahepatic LPV and the SMV will be visible, and the feasibility of completing the proximal and distal bypass attachments can be evaluated. The positive rate of the SMV is 100%. The Rex recess is an ideal location for placement of the shunt because it is rarely involved with cavernous transformations and collaterals [5].

Cárdenas et al. [16] reported that a Rex recess of $\ge$ 2 mm is the main indication for MRB and accounted for 63.2% of the cases. A total of 26.3% of children were able to undergo MRB even when the Rex recess was < 2 mm. For this reason, for the first time, we divided the CT manifestations of the Rex recess of all children with EHPVO into four groups. Children with types 1 and 2 could undoubtedly undergo MRB. Patients with a Rex recess of < 2 mm or even those displaying segment III PV in CT may also be suitable for MRB. Poor display of the Rex recess may be related to insufficient filling of the PV because of portal hypertension. The type 3 classification increased the CT positive rate from 63.2% to 75.0%. In type 4, there is complete loss of PV landmarks, and the incidence of type 4 was about 25%. However, we found that 55.6% of children with type 4 could still undergo MRB and need further evaluation. Chaves et al. [17] preferred WHVP to improve visualization of the intrahepatic portal venous system. Previously, children with a type 2 to 4 Rex recess required WHVP to confirm the presence of the Rex recess, meaning that 90.8% of children required interventional surgery. We recommend that only children with type 4 require WHVP. A total of 16.7% of patients underwent a change in their CT classification, suggesting that the progression of the disease may be a factor that affects the display of the Rex recess. Three notable findings of this study are as follows. First, as long as the Rex recess is displayed in any CT examination before surgery, MRB may be successful. Second, WHVP is required only when the Rex recess and its branch are not visible because most of these children can also undergo MRB. Third, the Warren procedure may not affect the display or CT classification of the Rex recess.

In clinical practice, the Rex recess may be confused by collaterals running alongside it or a dilated left hepatic artery, especially in patients with a type 3 or 4 Rex recess. The 45- to 52-s portal venous phase may no longer be appropriate in children with EHPVO in our experience because of the poor display of the PVs in some of these children. We consider that the delayed phase may replace the portal venous phase in the future to reduce the amount of radiation.

In addition to the Rex recess and SMV, other relevant venous anatomical structures must be evaluated simultaneously. First, adequately sized splanchnic collateral vessels should be identified because they may be utilized for the meso-Rex graft instead of the internal jugular vein [11]. The left gastric (coronary) vein, inferior mesenteric vein, gastroepiploic vein, recanalized umbilical vein, and saphenous vein have been described as graft material [18–20]. Second, inspection of systemic veins, including the inferior vena cava and renal veins, is required. If an MRB candidate is found at surgery to have inadequate intrahepatic portal venous anatomy to support the graft, the next surgical option is typically the Warren procedure.

## Limitations

The neonatal history of most children was unclear. Additionally, the relationship between the long-term surgical effect (clinical symptoms and/or anastomotic stenosis) and type of Rex recess remains unknown. Finally, the study was retrospective in nature; we should use these diagnostic criteria for prospective studies.

## Conclusion

CT is a reliable method for visualization of the Rex recess in children with EHPVO. We recommend four categories of the Rex recess based on CT angiography. The sensitivity, specificity, PPV, NPV, and diagnostic accuracy of CT in evaluating the success of MRB were 100%, 83.8%, 42.1%, 100%, and 85.5%, respectively. Among the four types of Rex recesses on CT angiography, types 1 to 3 allow for the performance of MRB. Patients with a type 4 Rex recess should undergo WHVP before MRB.

## Data Availability

It is a retrospective diagnostic study performed at one institution. No complex statistical methods were necessary for this paper.

## Abbreviations

CT: Computed tomography
EHPVO: Extrahepatic portal vein obstruction
LPV: Left portal vein
MRB: Meso-Rex bypass
MRI: Magnetic resonance imaging
NPV: Negative predictive value
PPV: Positive predictive value
PV: Portal vein
RPV: Right portal vein
SMV: Superior mesenteric vein
SV: Splenic vein
WHVP: Wedged hepatic vein portography
